# Human Carboxylesterase 2 Reverses Obesity-Induced Diacylglycerol Accumulation and Glucose Intolerance

**DOI:** 10.1016/j.celrep.2016.12.070

**Published:** 2017-01-17

**Authors:** Maxwell A. Ruby, Julie Massart, Devon M. Hunerdosse, Milena Schönke, Jorge C. Correia, Sharon M. Louie, Jorge L. Ruas, Erik Näslund, Daniel K. Nomura, Juleen R. Zierath

**Affiliations:** 1 Section for Integrative Physiology, Department of Molecular Medicine and Surgery, Karolinska Institutet, 17177 Stockholm, Sweden; 2 Departments of Chemistry, Molecular and Cell Biology, and Nutritional Sciences and Toxicology, University of California, Berkeley, Berkeley, CA 94720, USA; 3 Molecular and Cellular Exercise Physiology Unit, Department of Physiology and Pharmacology, Karolinska Institutet, 17177 Stockholm, Sweden; 4 Division of Surgery, Department of Clinical Sciences, Danderyd Hospital, Karolinska Institutet, 17177 Stockholm, Sweden

**Keywords:** obesity, hepatic steatosis, carboxylesterase, lipidomics, insulin resistance, activity-based protein profiling, inflammation, diacylglycerol, serine hydrolase

## Abstract

Serine hydrolases are a large family of multifunctional enzymes known to influence obesity. Here, we performed activity-based protein profiling to assess the functional level of serine hydrolases in liver biopsies from lean and obese humans in order to gain mechanistic insight into the pathophysiology of metabolic disease. We identified reduced hepatic activity of carboxylesterase 2 (CES2) and arylacetamide deacetylase (AADAC) in human obesity. In primary human hepatocytes, CES2 knockdown impaired glucose storage and lipid oxidation. In mice, obesity reduced CES2, whereas adenoviral delivery of human CES2 reversed hepatic steatosis, improved glucose tolerance, and decreased inflammation. Lipidomic analysis identified a network of CES2-regulated lipids altered in human and mouse obesity. CES2 possesses triglyceride and diacylglycerol lipase activities and displayed an inverse correlation with HOMA-IR and hepatic diacylglycerol concentrations in humans. Thus, decreased CES2 is a conserved feature of obesity and plays a causative role in the pathogenesis of obesity-related metabolic disturbances.

## Highlights

• Obesity decreases hepatic activity of AADAC and CES2 in humans
• CES2 depletion impairs lipid and glucose metabolism in primary human hepatocytes
• Human CES2 expression reverses hepatic steatosis and glucose intolerance in mice
• CES2 controls a hepatic lipid network dysregulated in human and mouse obesity

## Graphical Abstract

**Figure:**
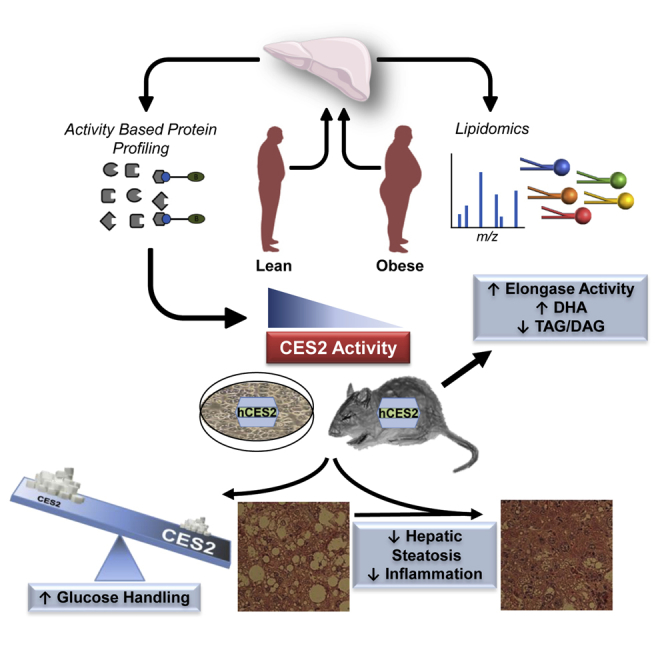

## Teaser

Ruby et al. utilize activity-based protein profiling to discover decreased arylacetamide deacetylase and carboxylesterase 2 activities in livers from obese humans. Carboxylesterase 2 controls a lipid network dysregulated in human obesity to reverse hepatic steatosis, glucose intolerance, and decrease inflammation in high-fat fed mice.

## Introduction

The prevalence of obesity has increased at an alarming rate over the past decades with dire public health consequences (NCD Risk Factor Collaboration, 2016). Excess weight dramatically increases a person’s risk of developing numerous diseases, including cardiovascular disease, cancer, and type 2 diabetes mellitus. Obesity-associated insulin resistance, characterized by intracellular defects in insulin action, is central to the etiology of related metabolic disturbances. Hepatic insulin resistance promotes aberrant glucose production, atherogenic dyslipidemia, and steatosis, which may lead to a hypercoagulable state and increased secretion of inflammatory proteins. Presently, effective therapies for hepatic insulin resistance are limited, with few clinical options available. The development of novel therapeutics requires an improved understanding of the mechanistic underpinnings of obesity and insulin resistance.

Transcriptomic and traditional proteomic technologies allow for the quantification of mRNA and protein levels, respectively. However, physiology is determined by enzyme activity, which is often tightly regulated by post-translational modifications that are undetectable by transcriptomic and standard proteomic analysis. Activity-based protein profiling (ABPP) was developed as a method to assess the functional state of enzyme families in native proteomes (Cravatt et al., 2008). ABPP utilizes site-directed chemical probes composed of a reactive group linked to a reporter tag for the detection of probe-bound enzymes. ABPP of serine hydrolase activity has proved particularly effective in the identification of dysregulated enzyme activities in pathological states and the development of small-molecule inhibitors (Nomura et al., 2010). For ABPP of serine hydrolases, a fluorophosphonate (FP) reactive group covalently binds the serine within the catalytic triad in an activity-dependent manner, and a biotin reporter tag enables enrichment of probe-bound enzymes for subsequent quantification by multidimensional protein identification technology (MudPIT) (Jessani et al., 2005). Thus, ABPP enables the simultaneous determination of multiple serine hydrolase activities from a single sample.

The serine hydrolase family consists of ∼240 enzymes, nearly evenly distributed between proteases and metabolic hydrolases, and regulates a wide array of physiological processes (Long and Cravatt, 2011). Numerous serine hydrolases impact metabolic homeostasis by controlling the bulk flux of metabolites, and the synthesis and degradation of key signaling molecules such as glucagon-like peptide-1 and the endocannabinoids (Blankman and Cravatt, 2013). Importantly, serine hydrolases have proved amenable to pharmacological intervention with six classes of drugs already approved for use in the clinic, including dipeptidyl peptidase-4 and pancreatic/gastric lipase inhibitors for the treatment of type 2 diabetes mellitus and obesity, respectively (Long and Cravatt, 2011).

Although ABPP has been used successfully to discover dysregulated enzymes in animal models of obesity, the activity levels of hepatic serine hydrolases in human obesity remain unknown (Barglow and Cravatt, 2004). Here, we utilized ABPP-MudPIT of serine hydrolases coupled to targeted lipidomics to gain a systems-level understanding of hepatic biochemical networks dysregulated in human obesity. ABPP revealed decreased activity of the metabolic serine hydrolases arylacetamide deacetylase (AADAC) and carboxylesterase 2 (CES2) in liver from obese humans. Utilizing loss- and gain-of-function studies in primary human hepatocytes and mice, respectively, we identify CES2 as a potent regulator of lipid metabolism, insulin sensitivity, and inflammation. Targeted lipidomics uncovers a CES2-regulated lipid network altered in human and mouse obesity. This work identifies dysregulated hepatic biochemical networks in human obesity and establishes CES2 as a regulator of metabolic disease.

## Results

### Altered Hepatic Lipids and Decreased Activity of AADAC and CES2 in Human Obesity

**Figure 1:**
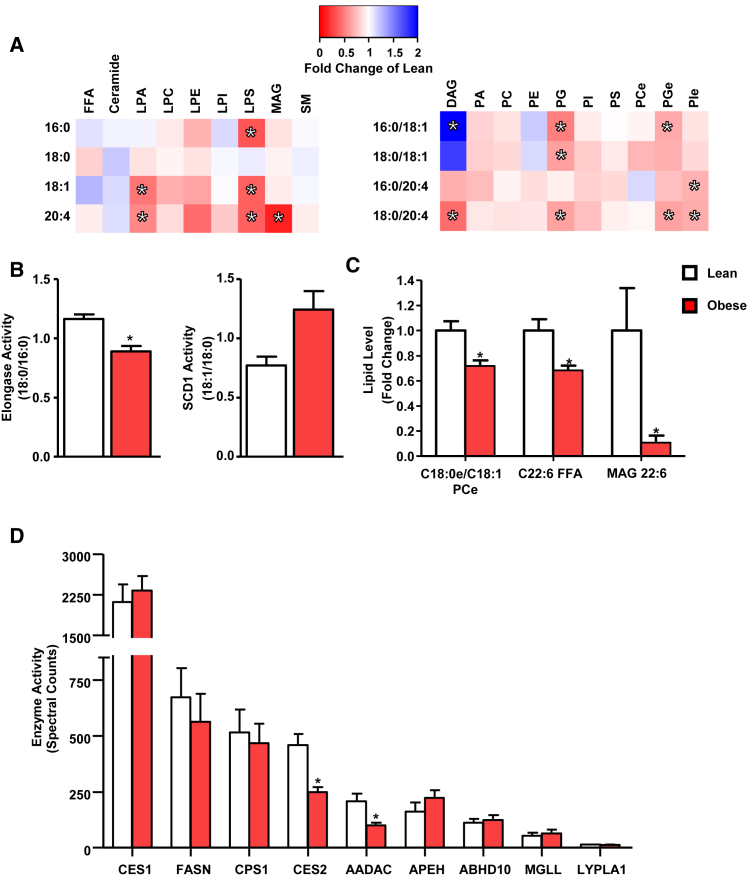
The Hepatic Lipidome and Serine Hydrolase Activities in Human Obesity (A) Single- and double-chain species detected within the targeted lipidomics from lean (n = 8) and obese (n = 15) individuals. Heatmaps are shown as fold change of obese compared to lean individuals. (B) Enzyme activities determined by product-to-precursor ratios from the targeted lipidomics dataset. (C) Additional lipids significantly altered by obesity. (D) Hepatic serine hydrolase activities in lean (n = 7) and obese (n = 9) individuals determined by ABPP-MudPIT. ^∗^q < 0.05. Data are presented as mean ± SEM.

Liver biopsies were obtained from lean (BMI 19–25) and obese (BMI 35–50) individuals during elective cholecystectomy or Roux-en-Y gastric bypass surgery, respectively. Despite marked obesity, insulin resistance, and reduced high-density lipoprotein (HDL) cholesterol, the obese individuals had neither diagnosed non-alcoholic fatty liver disease (NAFLD) nor elevated serum levels of liver enzymes (Table S1). To determine the effect of obesity on the liver lipidome, targeted lipidomics was used to quantify 116 individual lipid species. Obesity increased oleate-containing diacylglycerols (DAGs), whereas the ceramide and triglyceride (TAG) species tested were unchanged (Figure 1A and Data S1). Oleate-containing DAG concentrations correlated with HOMA-IR (r = 0.61 and 0.75 for DAG 16:0/18:1 and 18:0:18:1, respectively; both p < 0.01). Similar to changes observed in NAFLD, elongase and SCD1 activities were diminished and increased, respectively, although SCD1 activity failed to reach statistical significance (q = 0.09) (Figure 1B) (Kotronen et al., 2009).

DAGs containing arachidonic acid were diminished in obesity (Figure 1A). Decreases in arachidonyl-containing species were also observed in lysophospholipids and monoacylglycerol (MAG), whereas arachidonic acid remained unchanged (Figure 1A). Multiple forms of lysophosphatidylserine, lysophosphatidic acid, phosphatidylglycerol-ether, phosphatidylinositol-ether, and phosphatidylglycerol were lower in obese individuals (Figure 1A). Hepatic docosahexaenoic acid (DHA) and C22:6 MAG were also reduced in obesity (Figure 1C). The complete lipidomics dataset is available in Data S1.

To assess hepatic serine hydrolase activity in human obesity, ABPP-MudPIT was performed on liver biopsies with sufficient material (Table S1). Although the majority of serine hydrolases were unaffected by obesity, the activities of CES2 and AADAC were significantly reduced (Figure 1D).

### *CES2* Knockdown Impairs Glucose and Lipid Metabolism in Primary Human Hepatocytes

**Figure 2:**
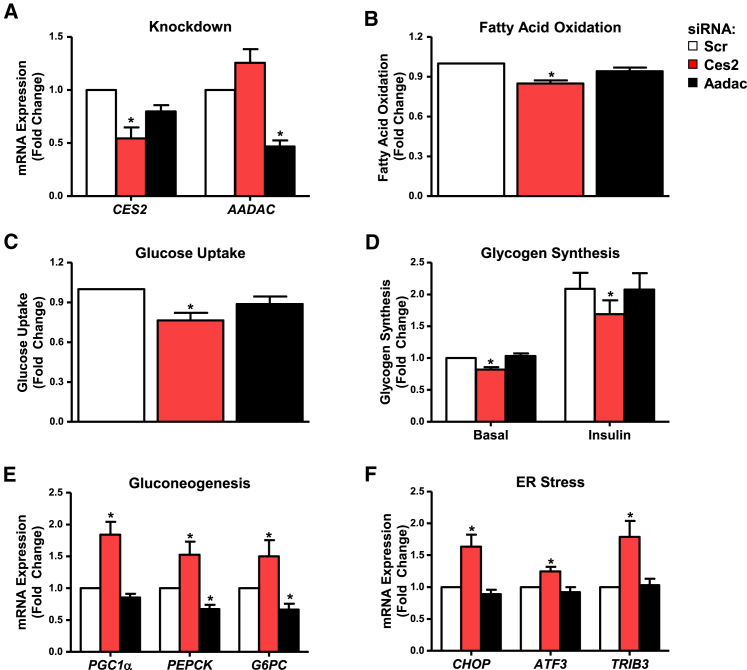
Metabolic Effects of *CES2* and *AADAC* Knockdown in Primary Human Hepatocytes (A) Knockdown of target genes in PHH 60 hr following reverse transfection with siRNA (n = 11). (B) Fatty acid oxidation in PHH (n = 9). (C) Uptake of ^3^H-deoxyglucose into PHHs in glucose-free medium (n = 6). (D) Incorporation of ^14^C-glucose into glycogen in PHH under basal and insulin-stimulated (120 nM) conditions (n = 6). (E and F) Gluconeogenic (E) and ER stress-responsive (F) gene expression in PHH (n = 11). ^∗^p < 0.05. Data are presented as mean ± SEM.

Although CES2 and AADAC are known to hydrolyze drugs and prodrugs, little is known about their role in energy metabolism. To determine whether decreased CES2 or AADAC impacts glucose and lipid metabolism, metabolic tracer studies were performed on small interfering RNA (siRNA)-treated primary human hepatocytes (PHHs). siRNA transfection reduced mRNA levels of *CES2* and *AADAC* by ∼50% (Figure 2A). Knockdown of *CES2* reduced fatty acid oxidation (Figure 2B). *CES2* knockdown diminished glucose uptake and incorporation into glycogen under both basal and insulin-stimulated conditions (Figures 2C and 2D). These effects were recapitulated with two additional independent siRNAs targeting *CES2* (data not shown). The expression of gluconeogenic and endoplasmic reticulum (ER) stress response genes was increased upon *CES2* knockdown (Figures 2E and 2F). *AADAC* knockdown had no effect on metabolic assays but decreased gluconeogenic gene expression (Figure 2). These data suggest that reducing *CES2* levels favors glucose output over uptake and lipid storage over oxidation.

### Decreased *Ces2* Levels in Genetic and Diet-Induced Murine Models of Obesity

To determine whether obesity alters CES2 function in mice, we determined the levels of *Ces2* isoforms in genetic and diet-induced murine models of obesity. Although humans have a single gene encoding *CES2*, mice have seven genes encoding isoforms of *Ces2* (Jones et al., 2013). To allow for absolute quantification across isoforms, isolated PCR amplicons were quantified and used to generate an internal standard curve. The major hepatic *Ces2* isoform, *Ces2a*, as well as the lower abundance *Ces2b*, *Ces2c*, and *Ces2g* isoforms were reduced in mice rendered obese by high-fat diet, mutation of leptin (ob/ob mice), or mutation of the leptin receptor (db/db mice) (data not shown). Interestingly, the murine *Ces2e* isoform is unaltered or increased in obesity (data not shown). The *Ces2f* and *Ces2h* isoforms were undetectable in mouse liver. Thus, decreased hepatic CES2 is a common feature of obesity in humans and multiple murine models.

### CES2 Reduces Adiposity and Improves Lipid Metabolism and Steatosis

**Figure 3:**
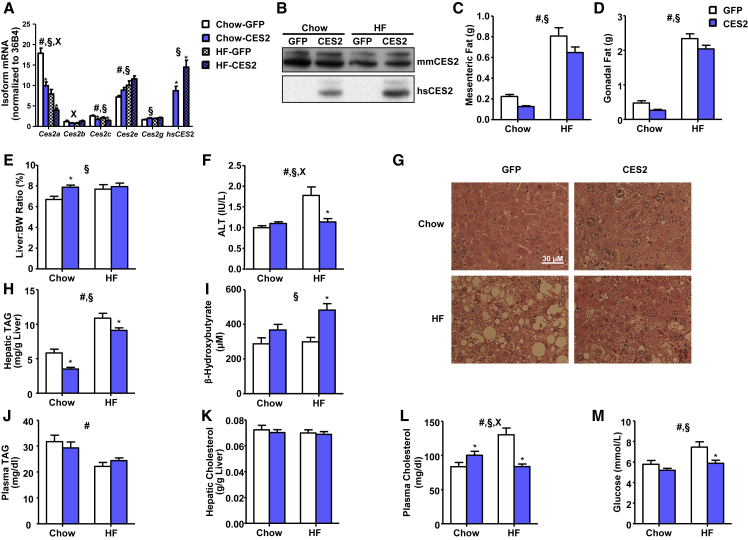
Effect of *CES2* Expression on Metabolic Parameters in Mice (A and B) Levels of human and mouse CES2 assessed by (A) qPCR and (B) western blot. (C–E) Mesenteric (C) and gonadal (D) adipose tissue mass and liver-to-body weight ratio (E) from dissection of 4-hr-fasted mice on day 8 following viral injection. (F) Serum ALT enzyme activity determined by enzymatic assay (n = 6–14). (G) Representative images of H&E staining of formalin-fixed tissue (n = 6). (H–M) Hepatic TAG (H), β-hydroxybutyrate (I), plasma TAG (J), hepatic cholesterol (K), plasma cholesterol (L), and plasma glucose (M) were determined by enzymatic end-point assays in plasma or hepatic extract (n = 12–17, unless otherwise noted). ^#^Diet effect; ^§^virus effect; ^x^interaction; ^∗^p < 0.05, Bonferroni post hoc test. Data are presented as mean ± SEM. See also Figure S1.

To determine whether ectopic *CES2* expression could reverse obesity-induced metabolic alterations, chow- and high-fat-fed mice were tail vein injected with a recombinant adenovirus encoding human CES2 or GFP. High-fat feeding decreased mRNA levels of *Ces2a* and *Ces2c*, and increased *Ces2e*, leading to a decrease in CES2 protein (Figures 3A and 3B). Human *CES2* mRNA was expressed at a level similar to the major endogenous mouse isoforms, and protein translation was verified by western blot (Figures 3A and 3B; Table S2). Addition of human *CES2* resulted in alterations of endogenous mouse isoforms, with profiles similar to those observed in obesity with lower *Ces2a* and *Ces2c* and higher *Ces2e* (Figure 3A).

*CES2* expression had no effect on body weight but reduced adipose tissue depots (Figures 3C, 3D, and S1A). Surprisingly, *CES2* administration increased liver weight, an effect especially pronounced in chow-fed mice (Figure 3E). The increased liver weight was not associated with alterations in serum ALT in chow-fed mice (Figure 3F). Remarkably, *CES2* expression completely reversed the high-fat diet-induced increase in serum ALT (Figure 3F). H&E staining revealed hepatocyte hypertrophy and associated eosinophilia in *CES2* mice (Figure 3G). *CES2* expression reversed high-fat feeding-induced hepatic steatosis (Figure 3G). This was confirmed at the biochemical level by a reduction in hepatic TAGs (Figure 3H). Alterations in hepatic TAGs were coincident with decreased expression of lipogenic genes and increased serum levels of β-hydroxybutyrate (Figures 3I and S1B). As hepatic hypertrophy with lower TAG content and increased ketone body production is suggestive of PPARα activation, we determined the mRNA level of *Pparα* and its target genes (Pawlak et al., 2015). Despite modest elevations of *Pparα* itself, the levels of canonical PPARα target genes were unchanged or decreased by *CES2* expression, suggesting that increased PPARα signaling is not responsible for the observed hepatic hypertrophy (Figure S1C).

Plasma TAGs and hepatic cholesterol were unaffected by *CES2* expression (Figures 3J and 3K). *CES2* expression increased plasma cholesterol levels in chow-fed animals, but normalized diet-induced hypercholesterolemia (Figure 3L). *CES2* expression reversed diet-induced increases in glycemia (Figure 3M).

### CES2 Improves Glucose Metabolism

**Figure 4:**
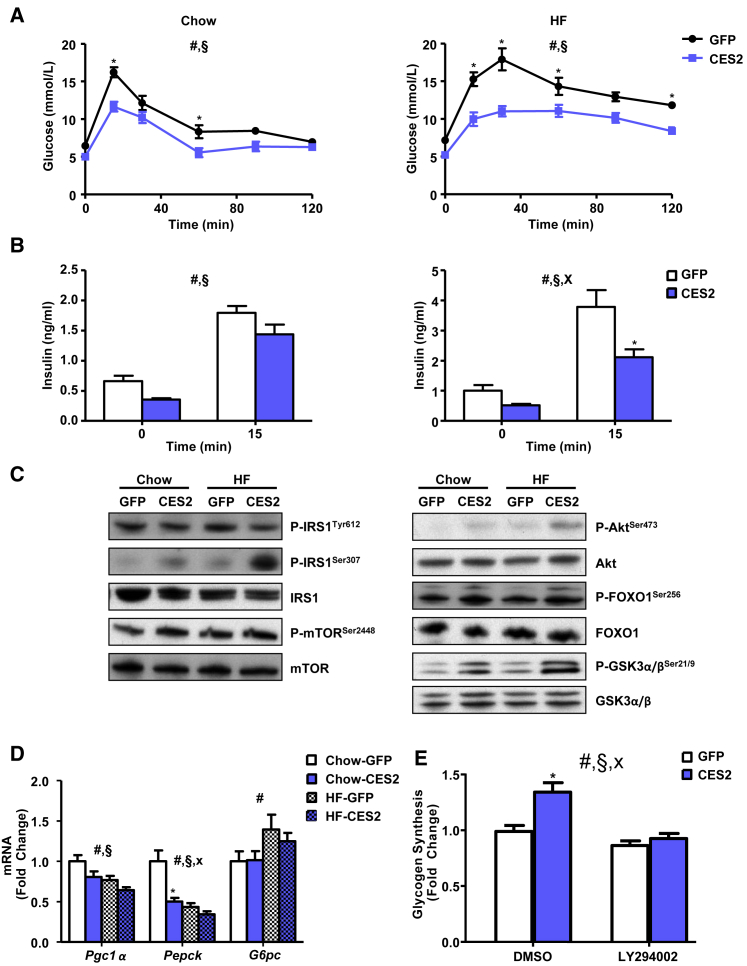
Effect of *CES2* Expression on Glucose Metabolism (A) On day 6 following viral injection, blood glucose response to an oral glucose load (2 g/kg) in 4-hr-fasted mice (n = 6–10). (B) Plasma insulin concentrations at basal and 15 min post oral glucose load (n = 6–10). (C) Western blot analysis of phosphorylation of the insulin signaling pathway in livers from 4-hr-fasted mice on day 8 viral following injection (n = 12–17). (D) qPCR analysis of gluconeogenic genes (n = 12–17). (E) Incorporation of ^14^C-glucose into glycogen in adenovirus-transduced primary mouse hepatocytes with 3-hr pretreatment of DMSO or LY294002 (10 μM) (n = 6). ^#^Diet or time effect; ^§^virus effect; ^x^interaction; ^∗^p < 0.05, Bonferroni post hoc test. Data are presented as mean ± SEM. See also Figure S2.

As *CES2* expression improved fasting glycemia, we performed an oral glucose tolerance test to assess the impact of *CES2* on glucose handling. Expression of *CES2* improved glucose tolerance in chow- and high-fat-fed mice (Figure 4A). *CES2* mice required less insulin under both basal and glucose-stimulated conditions to achieve improved glycemic control, suggesting improved insulin sensitivity (Figure 4B). Consistent with a lower requirement for insulin, hepatic phosphorylation of key components of the insulin signaling pathway (Akt, mTOR, FOXO1, and GSK3α/β) was increased by *CES2* expression (Figure 4C; Table S2). In line with increased phosphorylation and subsequent nuclear exclusion of FOXO1, the expression of gluconeogenic genes was decreased (Figure 4D). IRS1 phosphorylation at Ser307 was increased by *CES2* administration upon high-fat feeding, whereas tyrosine phosphorylation of IRS1 was decreased (Figure 4C; Table S2). Thus, *CES2* improves glucose tolerance, lowers insulin concentrations, and stimulates Akt signaling.

To test whether CES2 regulation of glucose metabolism is cell autonomous, primary mouse hepatocytes were transduced with adenoviruses encoding GFP or CES2. CES2 expression decreased expression of gluconeogenic genes and increased glucose incorporation into glycogen (Figures 4E and S2A). To determine whether CES2 effects were dependent on PI3 kinase, transduced hepatocytes were treated with the PIK3 kinase inhibitor LY294002. LY294002 reversed CES2-induced increases in Akt signaling and glucose incorporation into glycogen (Figures 4E and S2B). Thus, CES2 stimulates Akt signaling in a PI3 kinase-dependent manner to control hepatocyte glucose metabolism.

### CES2 Suppresses Hepatic Inflammation despite Increased JNK and IKK Phosphorylation

**Figure 5:**
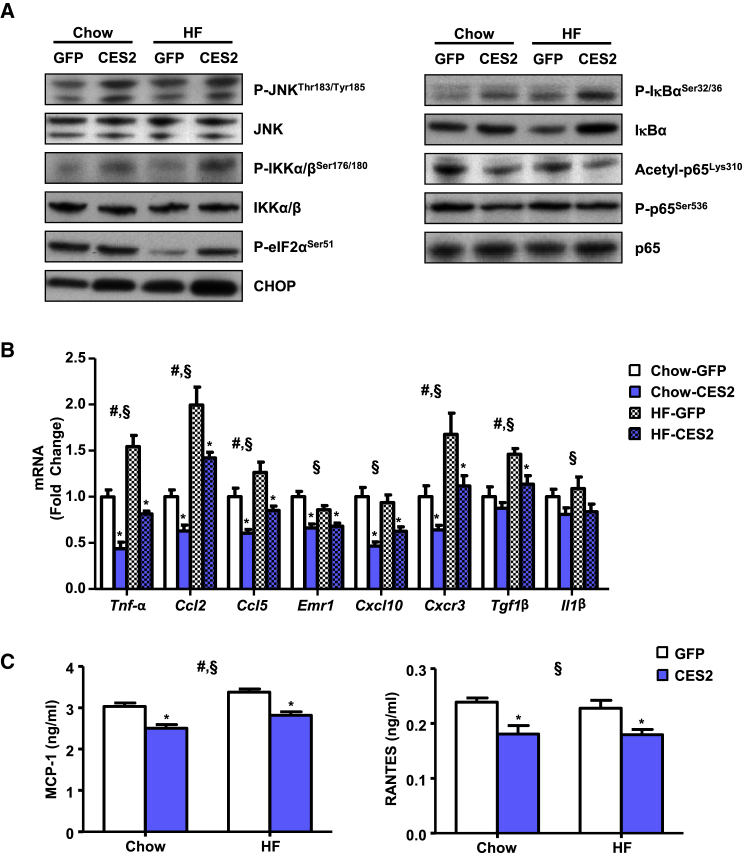
Effect of *CES2* Expression on Hepatic Inflammation (A) Western blot analysis of inflammatory signaling proteins. (B) Expression of cytokine mRNA by qPCR. (C) MCP-1 and RANTES concentration by ELISA (n = 12–17). ^#^Diet effect; ^§^virus effect; ^∗^p < 0.05, Bonferroni post hoc test. Data are presented as mean ± SEM. See also Figure S2.

Although the effect of IRS1 ser307 phosphorylation on insulin sensitivity is controversial, JNK and IKK are known to target this site (Ozcan et al., 2006). JNK and IKK phosphorylation was increased by *CES2* expression and high-fat diet (Figure 5A). As CES2 is an ER-resident lipase and ER stress is known to induce both IKK and JNK, we determined the levels of the ER stress markers p-eIF2α and CHOP. Both high-fat feeding and *CES2* expression increased protein levels of CHOP (Figure 5A). eIF2α phosphorylation was decreased by diet-induced obesity, and *CES2* expression reversed this effect (Figure 5A).

As IKK regulates inflammation, western blot analysis for components of the NF-κB pathway was performed. Consistent with increased IKK activity, IκBα phosphorylation was increased in an additive manner by high-fat diet and *CES2* (Figure 5A; Table S2). IκBα phosphorylation is normally associated with subsequent degradation of IκBα. Paradoxically, *CES2* expression leads to increased levels of IκBα despite increased phosphorylation (Figure 5A; Table S2). Consistent with increased stability of IκBα, phosphorylation and acetylation of the p65 subunit of NF-κB were decreased in *CES2*-expressing mice (Figure 5A; Table S2). To determine whether changes in phosphorylation and acetylation of p65 led to alterations in gene expression, the mRNA levels of NF-κB-regulated cytokines were assessed. *CES2* expression decreased mRNA levels of a broad array of inflammatory cytokines (Figure 5B). Similar to mRNA, hepatic protein levels of MCP-1 and RANTES, encoded by *Ccl2* and *Ccl5*, respectively, were diminished by *CES2* expression (Figure 5C). To determine whether the effects are cell autonomous, primary mouse hepatocytes were transduced with adenovirus encoding GFP or CES2. CES2 administration increased phosphorylation of the IKK targets, Ser307 on IRS1 and IκBα, but retained increased levels of IκBα (Figure S2C). CES2 expression reduced LPS-stimulated expression of *Tnf-α* and *Ccl2* (Figure S2D). Thus, despite increased activity of IKK and JNK, *CES2* expression decreases activation of NF-κB and levels of inflammatory cytokines.

### CES2 Is a TAG/DAG Lipase That Reverses Components of the Obese Lipidome

**Figure 6:**
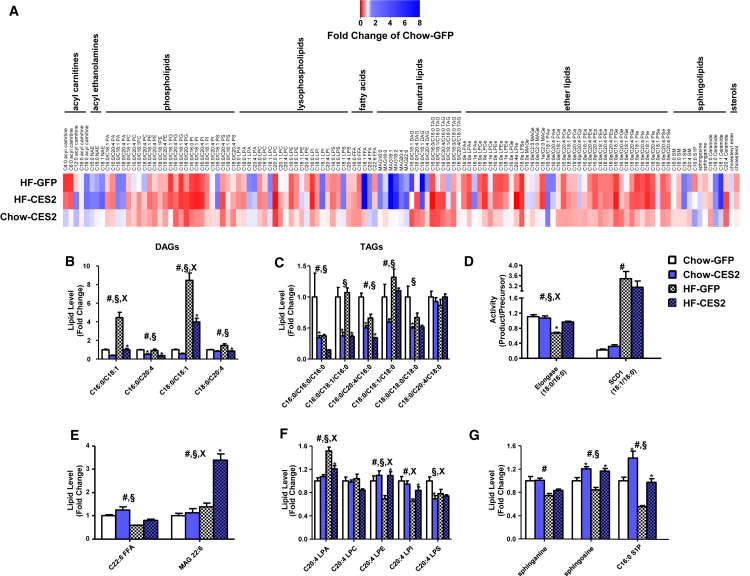
Effect of *CES2* Expression on the Hepatic Lipidome (A) Heatmap of all lipids within the targeted lipidomic analysis presented as a fold of chow-GFP. (B–G) Specific lipid classes of interest are highlighted in DAG (B), TAG (C), enzyme activities (D), DHA (E), arachidonyl-containing lysophospholipids (F), and sphingolipids (G) (n = 5–6). All main effects were corrected for multiple testing. ^#^Diet effect; ^§^virus effect; ^x^interaction; ^∗^p < 0.05, Bonferroni post hoc test. Data are presented as mean ± SEM. See also Figures S3 and S4.

As CES2 is a metabolic serine hydrolase, we sought to investigate the impact of *CES2* expression on the hepatic lipidome. *CES2* expression led to widespread changes in phospholipids, lysophospholipids, and ethers lipids, which were comparable to those induced by high-fat diet feeding (Figures 6A and S3). Similar to our observations in human obesity, high-fat diet feeding robustly increased oleate-containing DAGs, and this effect was reversed by *CES2* expression (Figure 6B). More broadly, *CES2* dramatically decreased levels of nearly all DAG and TAG species tested (Figures 6B and 6C). To test whether CES2 possesses intrinsic lipase activity, TAG and DAG hydrolysis was determined in microsomes isolated from High Five insect cells expressing human CES2. Human CES2-containing microsomes had increased hydrolytic rates for TAG and DAG (Figure S4). As DAGs are known to regulate insulin sensitivity through the activation of protein kinase C (PKC), we assessed the intracellular localization of PKCε. Although diet-induced obesity produced the expected increase in PKCε translocation to the membrane, *CES2* was without effect (Figure S4C).

As in human obesity, high-fat feeding decreased levels of DHA and dysregulated SCD1 and elongase activities (Figures 6D and 6E). *CES2* expression increased levels of DHA, and when combined with high-fat feeding, robustly increased DHA-containing MAG (Figure 6E). Although SCD1 activity was unaffected, the diet-induced decrease in elongase activity was reversed by *CES2* expression (Figure 6D). Obesity in mice, similar to humans, decreased arachidonyl-LPE, LPI, and LPS (Figure 6F). High-fat feeding increased arachidonyl-LPA, and *CES2* expression reversed obesity-induced alterations in arachidonyl-LPA, LPI, and LPE (Figure 6F). Sphingosine and C16:0 S1P were decreased by obesity and increased by CES2 expression (Figure 6G). The complete mouse lipidomics dataset is available in Data S2. Overall, we identified lipids altered by obesity in humans and mice that are regulated by CES2.

### CES2 Activity Correlates with Insulin Resistance and Hepatic DAG Content in Humans

As our data suggest that CES2 is an important regulator of insulin sensitivity and levels of DAG in obese mice, we investigated whether CES2 activity associated with these parameters in humans. Indeed, CES2 activity displayed a strong inverse correlation with HOMA-IR and oleate-containing DAG species (Figure S4). By contrast, AADAC activity did not significantly associate with HOMA-IR nor with DAG species (Figure S4). These data suggest that CES2 influences insulin sensitivity and regulates hepatic lipid metabolism in human obesity.

## Discussion

Despite the central role of liver in metabolic health, studies in humans are limited by tissue availability and are often restricted to comparisons between obese individuals with and without NAFLD (Allard et al., 2008, Gorden et al., 2015, Kotronen et al., 2009, Puri et al., 2007). Here, we studied liver biopsies from otherwise healthy lean individuals undergoing elective cholecystectomy and obese individuals without known NAFLD to gain insight into the early events in disease progression. Collectively, our results map the landscape of hepatic lipids and serine hydrolase activities in human obesity and identify decreased CES2 as a driving force of obesity-induced metabolic disease.

Animal studies emphasize roles for DAGs and ceramides as major lipotoxic species promoting metabolic disease in obesity (Neuschwander-Tetri, 2010). The unaltered hepatic ceramide concentrations in human obesity observed in our study and in NAFLD, challenge the relevance of hepatic ceramides to human metabolic disease (Kotronen et al., 2009). In contrast, our findings that hepatic monounsaturated DAGs are increased and correlated with HOMA-IR further highlight the role of DAGs in humans (Kotronen et al., 2009, Kumashiro et al., 2011, Puri et al., 2007). Arachidonic acid was decreased in multiple lipid species, including DAGs, in obesity. The decreases in polyunsaturated fatty acids across many lipid species observed here and in NAFLD have been attributed to defects in elongation and desaturase activities as the essential fatty acid precursors appear unaltered in NAFLD (Allard et al., 2008, Araya et al., 2004, Puri et al., 2007). In our study, arachidonic acid was unaltered, suggesting that incorporation into lipid species rather than supply may be altered by obesity. Similar to NAFLD, obesity altered elongase and SCD1 activity indexes (Kotronen et al., 2009). The finding that lipidomic changes in obesity closely mirror those observed in NAFLD suggest these alterations appear before the onset of NAFLD and progress on a spectrum of disease severity.

We identified alterations in specific lipid species not previously observed in NAFLD. Phosphatidylglycerols, phosphatidylglycerol-ethers, and lysophosphatidylserines were decreased in obesity. Although lysophosphatidylserine regulates peripheral glucose uptake and inflammation, its role in the liver remains unknown (Frasch and Bratton, 2012, Yea et al., 2009). Phosphatidylglycerol is produced exclusively in the mitochondria and is the precursor for cardiolipin (Morita and Terada, 2015). Interestingly, CGI-58 knockdown, which causes hepatic steatosis but prevents obesity and glucose intolerance, profoundly increases hepatic phosphatidylglycerol concentrations (Brown et al., 2010). Thus, decreased phosphatidylglycerols and phosphatidylglycerol-ethers may reflect alterations in mitochondrial content or function in livers from obese humans.

The major hepatic serine hydrolase activities, carboxylesterase 1 (CES1) and fatty acid synthase, were unaltered in human obesity. Unexpectedly, activity of CES2 and AADAC, enzymes that are best known for their role in the metabolism of xenobiotics, was decreased in livers from obese individuals. Adaptation of drug doses presents a challenge in obese patients, and the most appropriate method for dose correction remains controversial (Griggs et al., 2012). Our findings of altered levels of enzymes in drug breakdown and activation may inform determination of proper drug dosing in the context of obesity.

AADAC overexpression regulates lipid metabolism in McArdle-RH7777 hepatoma cells (Lo et al., 2010). Hepatoma cell lines have aberrant expression of drug-metabolizing enzymes that limits their use to such overexpression studies (Guo et al., 2011). PHH sustain expression of drug-metabolizing enzymes and are considered the gold standard for studying xenobiotic metabolism (Guo et al., 2011). Here, knockdown of endogenous AADAC in PHH did not alter fatty acid oxidation. However, our negative findings may be due to the modest level of AADAC knockdown and do not preclude a role for this enzyme in metabolism.

Hepatic lipid content reflects the balance of lipid synthesis and removal. In PHH, CES2 promotes lipid oxidation in a cell-autonomous manner. Decreases in TAG content, reversal of histologically determined steatosis, and increases in serum ketone bodies suggest that human CES2 plays a similar role in vivo. Increased β-oxidation was independent of transcriptional activation of this pathway, suggesting that CES2 could play a more direct role. In addition to apparent increases in oxidation, CES2 decreased expression of lipogenic genes. Increases in polyunsaturated fatty acids, such as DHA, inhibit SREBP1c and subsequent expression of lipogenic genes (Deng et al., 2002, Xu et al., 2001). Similar to CES1, CES2 may regulate DHA levels to inhibit SREBP1c (Quiroga et al., 2012). Lipidomics revealed robust decreases in TAG and DAG, and CES2 displayed significant TAG and DAG hydrolase activities. Although the role of arachidonic acid containing lysophospholipids is unknown, our observation that they are decreased in human and murine obesity and reversed by CES2 raises interest in their role in hepatic metabolism. The elongase activity index was also decreased in both human and murine obesity and reversed by CES2. In mice, ELOVL6 performs the reaction measured by the elongase activity index. ELOVL6 knockout increases hepatic lipid accumulation with inconsistent effects on body weight and insulin sensitivity (Matsuzaka et al., 2007, Moon et al., 2014). This suggests that reversing the diet-induced decrease in elongase activity may contribute to the reversal of hepatic steatosis upon CES2 administration.

ER stress sits at the intersection of inflammation and metabolic disease (Hotamisligil, 2010). Interestingly, both loss of CES2 in vitro and addition of CES2 in vivo stimulated ER stress. As CES2 is an ER-resident enzyme, appropriate levels of CES2 may be necessary to maintain ER homeostasis. In support of this notion, CES2 administration decreased an extensive array of phospholipids and ether lipids, similar to alterations observed in diet-induced obesity, which may be responsible for triggering ER stress. JNK and IKK are key kinases in transmitting the signals from ER stress to promote insulin resistance and inflammation (Hotamisligil, 2010). Remarkably, CES2 dissociates activation of ER stress, IKK, and JNK from inflammation and insulin resistance.

Hepatic inflammation contributes to perturbations in metabolic homeostasis and causes the progression from simple steatosis to more serious liver diseases. Despite activation of IKKα/β and increased phosphorylation of IκBα, CES2 administration had an anti-inflammatory effect associated with reduced expression of NF-κB target genes. This paradox exists due to continued increases in IκBα despite its phosphorylation. Increases in DHA, which decreases p65 nuclear translocation, may also contribute to the anti-inflammatory effect of CES2 (Mullen et al., 2010). The anti-inflammatory effects of CES2 overexpression suggest that decreased CES2 may promote the progression from NAFLD to non-alcoholic steatohepatitis (NASH).

In our studies, CES2 emerged as a powerful regulator of glucose metabolism both in vitro and in vivo. Interestingly, CES2 appears to affect both insulin-dependent and -independent pathways. The improvement in glucose tolerance, with decreased serum insulin and activation of Akt, suggests enhanced insulin sensitivity in CES2 mice. Lipid accumulation and inflammation are thought to underlie insulin resistance. CES2 dramatically decreased hepatic levels of DAGs, which stimulate PKC enzyme activity to induce insulin resistance. Despite this decrease in DAG, CES2 did not alter PKCε activation upon high-fat feeding. The reasons for this are unclear, but may reflect the cellular localization of DAG (Bergman et al., 2012). Upon CES2 administration, we observed increased activity of IKK and JNK, which are thought to induce insulin resistance through phosphorylation of serine sites on IRS1 and IRS2 (Giraud et al., 2004, Hotamisligil, 2010). Although Ser307 on IRS1 has gained particular attention in promoting insulin resistance, it is phosphorylated in response to insulin and a knockin mutation that prevents phosphorylation promotes, rather than prevents, insulin resistance (Copps et al., 2010, Giraud et al., 2004). However, in our study, Ser307 phosphorylation was associated with decreased tyrosine phosphorylation of IRS1. This suggests that IRS2 may be responsible for activation of Akt and downstream signaling. Alternatively, Akt could be stimulated independent of insulin, reducing the requirement for circulating insulin concentrations. In support of this, CES2 knockdown decreased glucose uptake and incorporation into glycogen in vitro in the absence of insulin. Moreover, in insulin-sensitive fasted chow-fed animals, CES2 further increased Akt signaling while reducing serum insulin. In primary mouse hepatocytes, CES2 administration stimulated Akt signaling and glucose incorporation into glycogen in a PI3 kinase-dependent manner. Interestingly, sphingosine and C16:0 S1P species were elevated in CES2 mice. S1P activates Akt signaling through PI3 kinase by activating its receptors in various settings and plays a role in downstream effects of the adiponectin receptors (Holland et al., 2011, Osawa et al., 2001). In vivo, hepatic sphingosine kinase 2 or acid sphingomyelinase overexpression increase S1P, glucose tolerance, and Akt signaling independent of IRS1/2 (Lee et al., 2015, Ma et al., 2007). Taken together, this raises the intriguing possibility that CES2 regulates sphingolipid metabolism to control hepatic glucose metabolism.

Overall, CES2 improves glucose tolerance and decreases the requirement for serum insulin, despite increased activation of IKK and JNK and aberrant phosphorylation of IRS1. In the setting of CES2 overexpression, as in conditional XBP1 knockout mice fed a fructose diet, improving lipid homeostasis reverses glucose intolerance despite elevated ER stress (Jurczak et al., 2012). The strong correlation between hepatic CES2 activity and HOMA-IR suggests that our findings are pertinent to human insulin resistance in vivo.

While this manuscript was in preparation, Li et al. (2016b) reported that mouse CES2 is a TAG hydrolase that prevents hepatic steatosis and that CES2 protein levels are decreased in patients with NASH. Similar to our findings with human CES2 expression, overexpression of mouse CES2 decreased adiposity, improved glucose tolerance, and reversal of steatosis (Li et al., 2016b). Despite these similarities, several differences exist between the overexpression of mouse and human CES2. Mouse CES2 overexpression reversed, whereas human CES2 expression activated ER stress (Li et al., 2016b). In contrast to our findings with human CES2, mouse CES2 overexpression activated PPARα and decreased levels of DHA (Li et al., 2016b). The divergent properties of human and mouse CES2 that underlie these phenotypic differences warrant further study.

In conclusion, CES2 is a robust and reproducible regulator of intermediary metabolism that is altered in mouse and human obesity. Mechanistically, CES2 promotes lipid oxidation to reverse hepatic steatosis and dissociates activation of ER stress, as well as the downstream effector proteins IKK and JNK, from inflammation, Akt activation, and glucose intolerance. Thus, decreased CES2 appears to be a causative factor in the progression of hepatic metabolic disease and suggests that strategies restoring CES2 activity may prove effective in the treatment of obesity-associated metabolic disease.

## Experimental Procedures

### Human Sample Collection

The Regional Ethics Committee of Stockholm approved this study. Participants provided informed written consent, and the study was performed in accordance with the Declaration of Helsinki. Liver biopsies were collected from 8 lean (BMI 19–25) individuals during elective cholecystectomy and from 15 obese (BMI 35–50) individuals during Roux-en-Y gastric bypass surgery. Biopsies were collected after an overnight fast at the beginning of the surgical procedure. Patients were not given intravenous glucose until the biopsies were obtained, and the obese subjects were not subjected to a preoperative diet. Biopsies were flash-frozen in liquid nitrogen.

### Lipidomic Analysis and Activity-Based Protein Profiling

Lipidomic analysis was performed as described (Louie et al., 2016). Identification and comparative quantitation of serine hydrolase activities from human liver proteomes by ABPP-MudPIT was conducted as previously described using FP-biotin (5 mM) (Jessani et al., 2005). Analysis between lean and obese individuals was limited to serine hydrolases detected in all samples.

### Primary Human Hepatocyte Isolation and Culture

Liver tissue was attained from the Liver Center, Karolinska University Hospital (Huddinge, Sweden), with informed donor consent from individuals with metastatic cancer or from donor livers unsuitable for transplantation. Primary human hepatocytes were isolated from freshly resected liver tissue as previously described (Li et al., 2016a). Cells were plated on collagen-coated plates in William’s-E media containing 25 mM HEPES, 2 mM glutamine, 120 nM insulin, and 100 nM dexamethasone, and transfected with lipofectamine RNAiMAX and 100 nmol of silencer select siRNA (Life Technologies) in 1% DMSO. Following 12-hr transfection, non-adherent cells were removed by washing with PBS and media replaced with William’s-E media with 1 nM insulin. All experiments were performed 48 hr after the end of transfection. Primary mouse hepatocytes were isolated, plated, and transduced as previously described (Correia et al., 2015).

### Radioactive Tracer Metabolic Assays

#### Fatty Acid Oxidation

Fatty acid oxidation was measured as previously described (Nascimento et al., 2015). Briefly, PHHs were incubated in media with radioactive ^3^H-palmitate (PerkinElmer) and cold palmitate (25 μM) in low-glucose DMEM for 3 hr. A portion of the media was transferred to charcoal slurry to bind un-metabolized palmitate. Following centrifugation, the amount of radioactivity associated with H_2_O was measured in the medium. The cells were washed with ice-cold PBS three times, and lysate was used for protein determination.

#### Glucose Uptake

2-Deoxyglucose uptake was measured as described (Al-Khalili et al., 2006). PHHs were serum starved for 4 hr in low-glucose DMEM. Following a PBS wash, glucose-free media with ^3^H-2-deoxyglucose (Moravek Biochemicals), and cold 2-deoxyglucose (10 μM) was added to the cells for 15 min. The assay was terminated by washing in ice-cold PBS four times. Cells lysate was analyzed for protein concentration and ^3^H content.

#### Glycogen Synthesis

Glycogen synthesis was determined as described (Nascimento et al., 2015). PHHs were serum starved in DMEM (low glucose) for 4 hr and stimulated with 120 nM insulin or vehicle control for 2 hr with addition of ^14^C-glucose (PerkinElmer) for the final 90 min. Cells were washed with ice-cold PBS three times and lysed. Protein concentration was determined in a portion of the lysate. Glycogen was precipitated in ethanol, isolated by centrifugation, washed, and suspended in water prior to analysis for radioactivity.

### qPCR

mRNA was extracted from cells and livers with the RNeasy Mini Kit (QIAGEN) and TRIzol reagent (Invitrogen), respectively, according to the manufacturer’s recommendations. RNA concentration was measured with Nanodrop 1000 (Thermo Scientific) and reverse transcribed using the High-Capacity cDNA RT Kit (Applied Biosystems). Relative quantitative real-time PCRs were performed in duplicate using SYBR Green reagents (Applied Biosystems). For absolute quantification of *Ces2* isoforms, PCR amplicons were isolated following gel electrophoresis, quantified, and diluted to form standard curves. Primer sequences are available in Supplemental Experimental Procedures.

### Animal Work

Male C57BL/6J mice (4 weeks old) were purchased from Charles River and housed for 1 week prior to diet treatment. Mice were maintained on a 12-h light/12-h dark cycle and received water and food ad libitum. Mice were fed with a standard rodent chow (4% fat, 16.5% protein, 58% carbohydrate, 3.0 kcal/g purchased from Lantmännen) or a high-fat diet (54.8% fat, 21.2% protein, 24% carbohydrates, 4.8 kcal/g from Envigo) for 16 weeks. Mice were injected with 1.6 × 10^9^ PFU particles of adenoviral vectors (Vector Biolabs) into the tail vein. Six days after injection, mice were fasted for 4 hr prior to an oral glucose tolerance test. Glucose was administrated orally to the mice (2 g/kg) and blood glucose was measured at 0, 15, 30, 60, 90, and 120 min (One-Touch Accu-Check Glucometer; Roche). Blood was collected at 0 and 15 min for insulin measurement by tail bleeding. Eight days post-injection, mice were fasted for 4 hr and anesthetized with Avertin (2.5% solution of 99% 2,2,2-tribromo ethanol and tertiary amyl alcohol; Sigma-Aldrich). Tissues were dissected, weighed, and immediately frozen in liquid nitrogen. All animal procedures were approved by the Regional Animal Ethical Committee (Stockholm, Sweden).

### Western Blotting

Western blot analysis was as described (Riedl et al., 2016). Antibodies used are provided in Supplemental Experimental Procedures. Ponceau S staining was used to confirm equal protein loading. Western blots were quantified by densitometry utilizing Quantity One Software (Bio-Rad), and quantifications are presented in Table S2.

### Biochemical Analysis

Cholesterol (Life Technologies) and triglyceride (Sigma-Aldrich) levels were determined in plasma and hepatic lipids extracted by iosproponal:heptane extraction (Massart et al., 2014). Serum ALT, β-hydroxybutyrate (Cayman Chemical), and insulin (Crystal Chem) levels were determined using commercial kits according to manufacturer instructions. Hepatic tissue was lysed in PBS containing 0.1% Tween 20 to analyze MCP1 (Biolegend) and RANTES (R&D Systems) levels by ELISA. Formalin-fixed livers were sent to HistoCenter AB for H&E staining.

### DAG and TAG Lipase Assays

Supersomes transduced with human CES2 (Corning; #453322) or negative control Supersomes (Corning; #456200) were utilized in TAG lipase activity assays as previously described (Ahmadian et al., 2011). DAG lipase activity assay was assessed with cold and ^14^C-labeled diolein. Reactions were terminated, and lipids were extracted and separated by thin-layer chromatography as previously described (Massart et al., 2014).

### Statistics

Statistical analyses were performed using GraphPad Prism 6.0 (GraphPad Software) and the q-value R package. A t test analysis with multiple testing correction was used to compare lean and obese individuals. For primary human hepatocyte experiments, data are presented as fold to account for inter-donor variation, and significance was assessed utilizing Friedman’s test with Dunn’s multiple-testing comparison. For animal experiments, two-way ANOVA was performed with a Bonferroni’s post hoc test within each dietary condition. Virus and interaction effects were subjected to multiple-testing correction for the murine lipidomics dataset. For all tests, significance was set at q or p < 0.05. Data are presented as mean ± SEM.

## Author Contributions

Conceptualization, M.A.R., D.K.N., and J.R.Z.; Investigation, M.A.R., J.M., D.M.H., M.S., J.C.C., and S.M.L.; Writing – Original Draft, M.A.R.; Writing – Review and Editing, M.A.R, D.K.N., and J.R.Z.; Visualization, M.A.R. and J.M.; Funding Acquisition, M.A.R., E.N., D.K.N., and J.R.Z.; Resources, J.L.R., E.N., D.K.N., and J.R.Z.; Supervision, D.K.N. and J.R.Z.

## Supplemental Information

Document S1. Supplemental Experimental Procedures, Figures S1–S4, and Tables S1 and S2

Data S2

Document S2. Article plus Supplemental Information
