# Supplementary material for: Human Carboxylesterase 2 Reverses Obesity-Induced Diacylglycerol Accumulation and Glucose Intolerance
Source: Cell Rep. 2017 Jan 17;18(3):636–46. doi: 10.1016/j.celrep.2016.12.070 (PMC5276805; doi:10.1016/j.celrep.2016.12.070)
Supplement: Document S1. Supplemental Experimental Procedures, Figures S1–S4, and Tables S1 and S2 [file mmc1.pdf]

Cell Reports, Volume *18*

Supplemental Information

Human Carboxylesterase 2 Reverses Obesity-Induced Diacylglycerol Accumulation and Glucose Intolerance

Maxwell A. Ruby, Julie Massart, Devon M. Hunerdosse, Milena Schönke, Jorge C. Correia, Sharon M. Louie, Jorge L. Ruas, Erik Näslund, Daniel K. Nomura, and Juleen R. Zierath

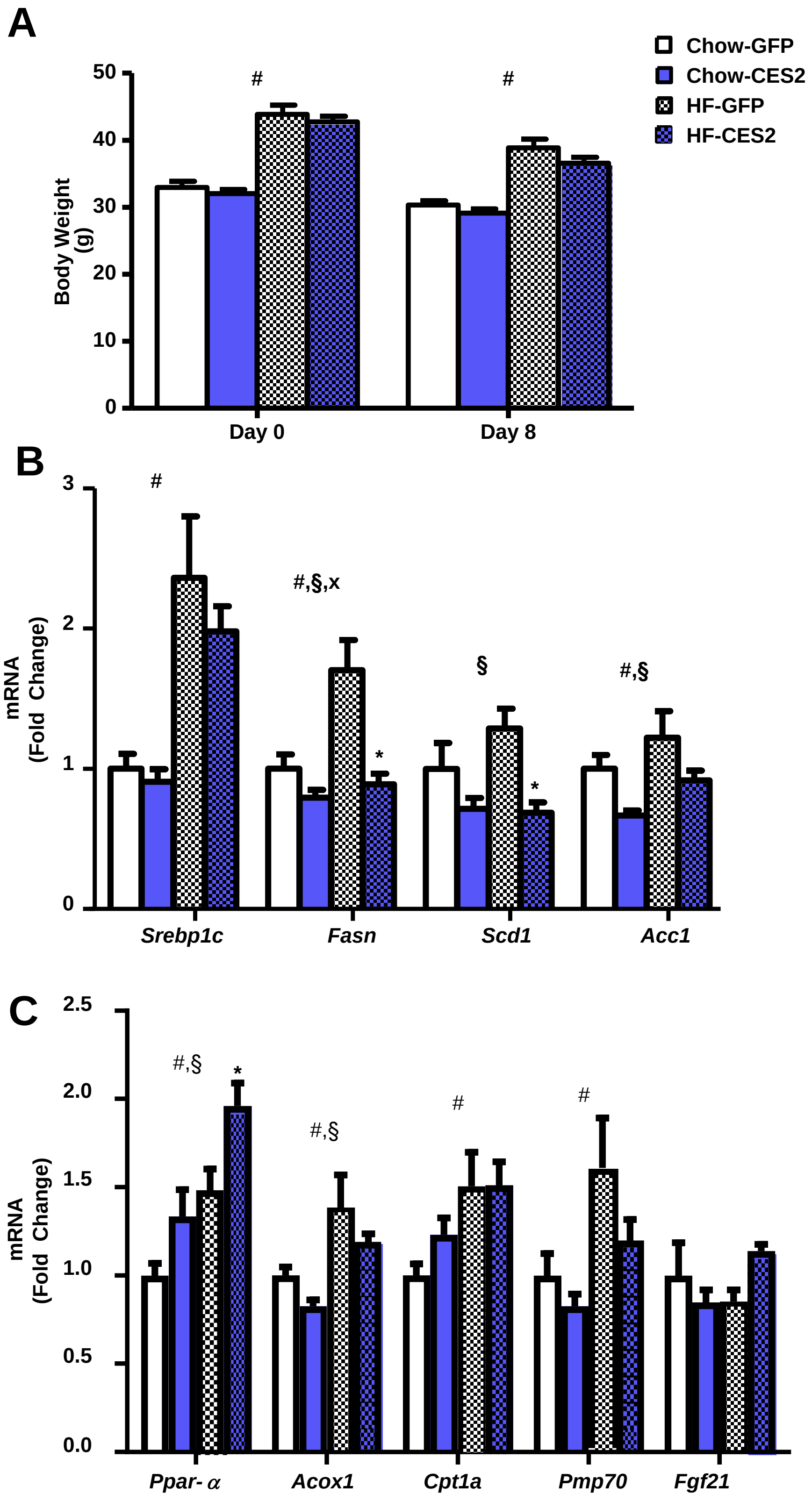

Supplemental Figure 1. Body Weight and Hepatic Expression of SREBP1c and PPARα Target Genes in Mice Injected with Ces2 Adenovirus. Related to Figure 3. (A) Body weights of *ad libitum* fed mice on day 0 and 4 h fasted mice on day 8. qPCR analysis of (B) SREBP1c, (C) PPARα and their target genes (n=12-17). #diet effect, §virus effect, x interaction, *p<0.05 Bonferroni *post hoc* test.

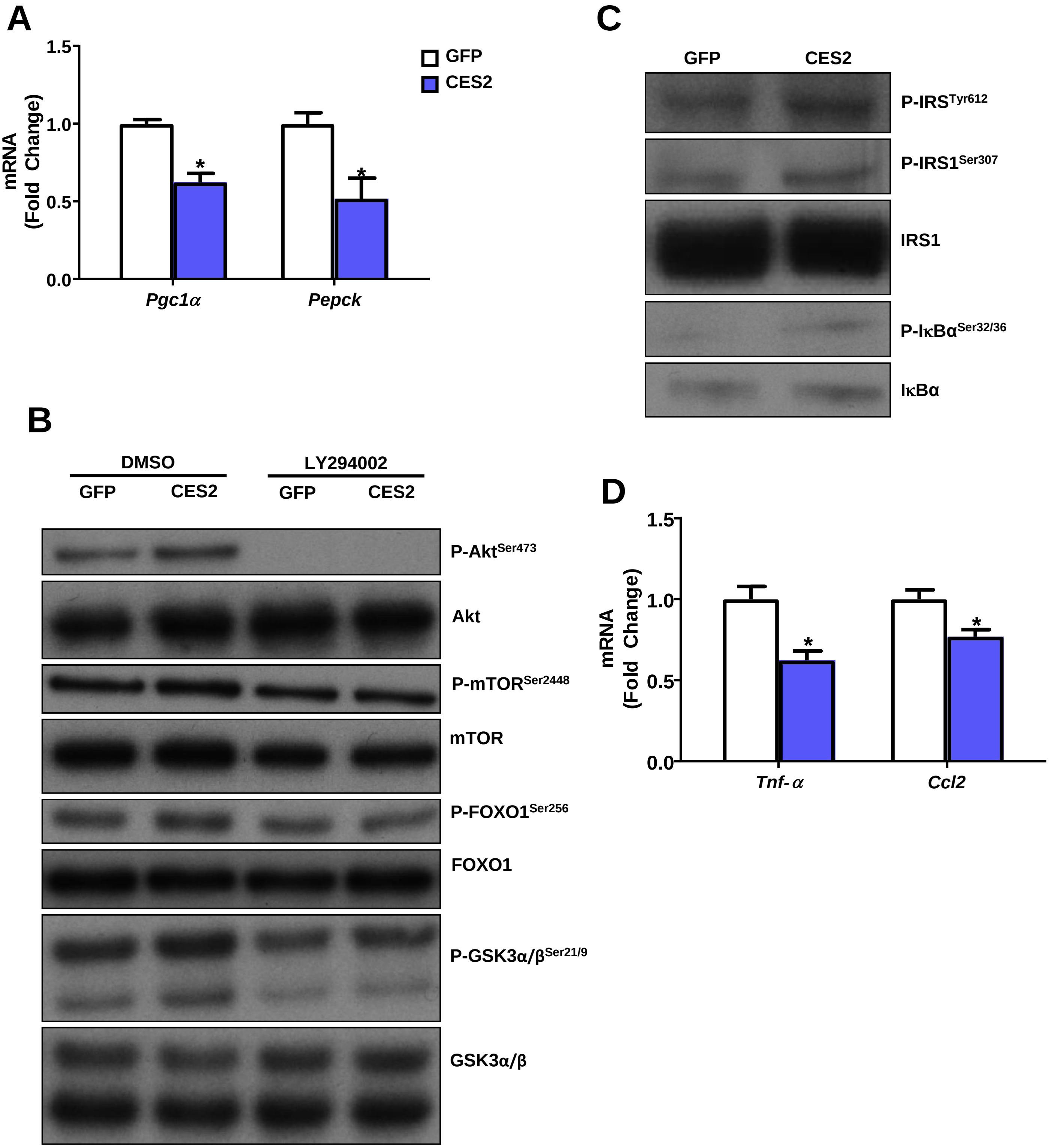

Supplemental Figure 2. Cell Autonomous Effects of CES2 in Primary Mouse Hepatocytes. Related to Figures 4 and 5. qPCR analysis of (A) gluconeogenic genes in mouse primary hepatocytes transduced with GFP or CES2 (n=6). Representative western blots of transduced mouse primary hepatocytes (B) treated with DMSO or LY294002 (10 µM) for 3 h or (C) untreated hepatocytes (n=6). qPCR analysis of LPS-stimulated (100 ng/ml; 4 h) (D) inflammatory gene expression in primary mouse hepatocytes (n=6). $^*p<0.05$.

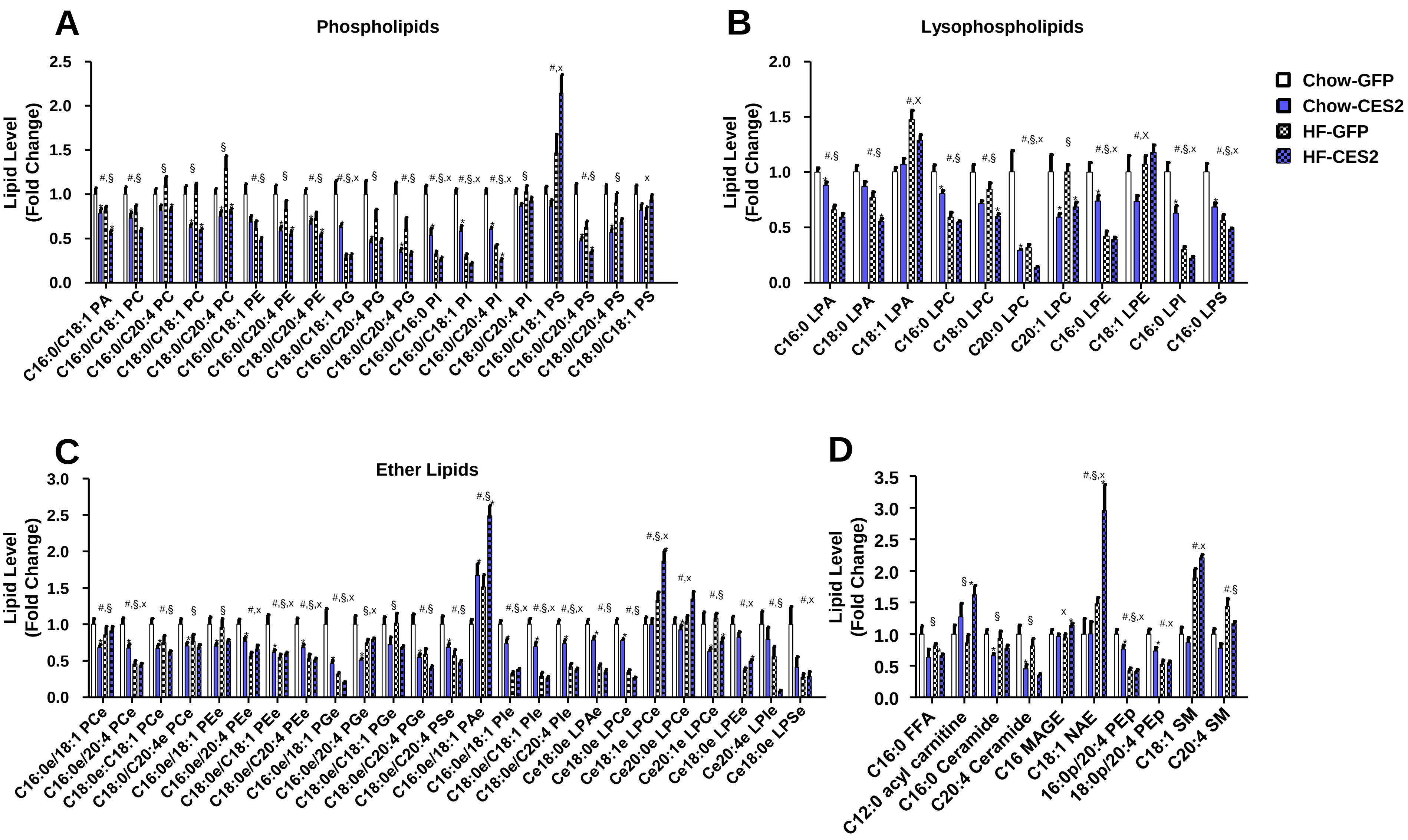

Supplemental Figure 3. Additional Lipids Significantly altered by CES2 administration. Related to Figure 6. (A) Phospholipids, (B) Lysophospholipids, (C) Ether Lipids and (D) miscellaneous lipids with significant virus or interaction effects (n=5-6). All main effects were corrected for multiple testing. #diet effect, §virus effect, Xinteraction, *p<0.05 Bonferroni *post hoc* test.

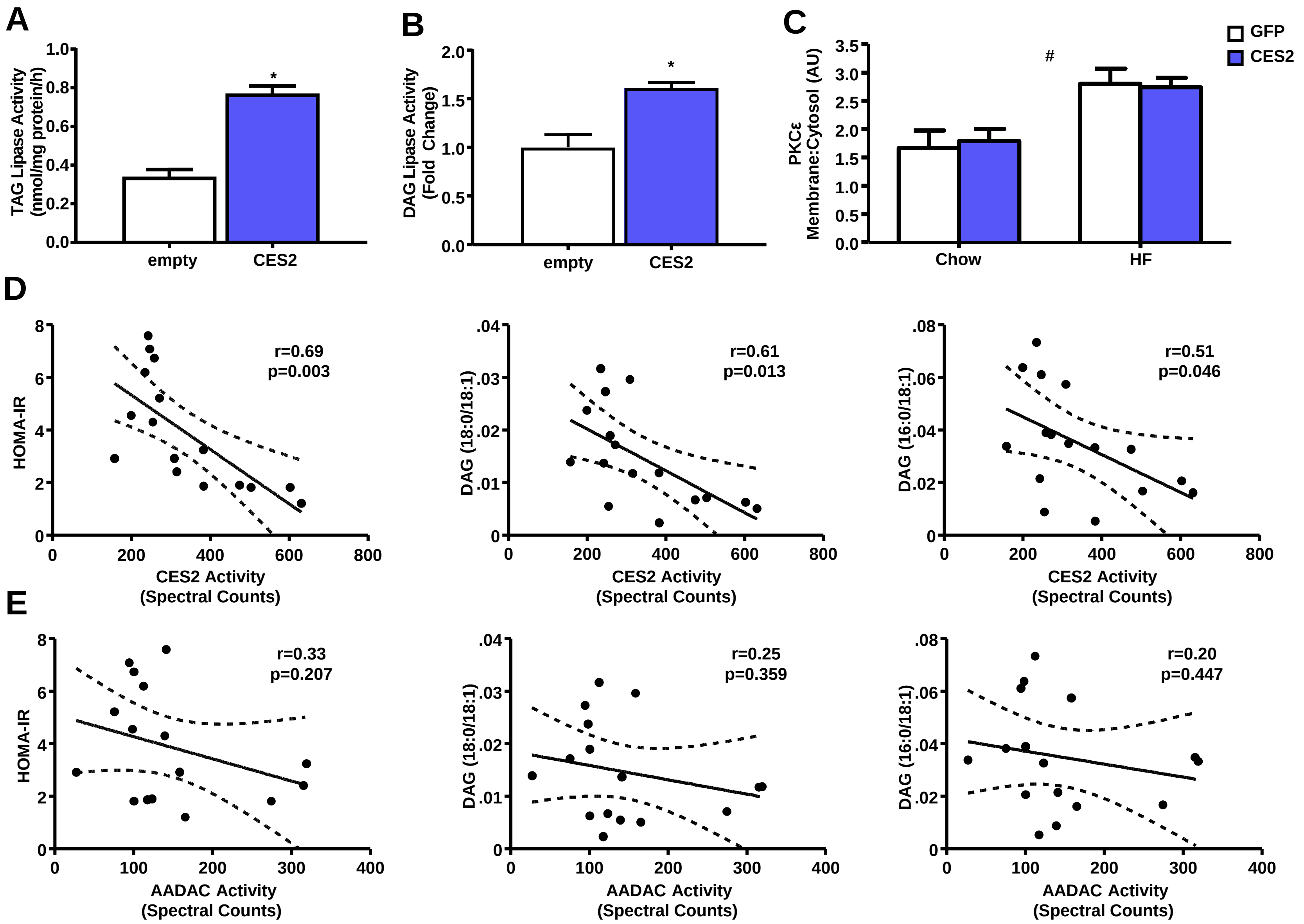

Supplemental Figure 4. CES2 is a TAG and DAG hydrolase whose Activity Correlates with HOMA-IR and DAG concentrations in Humans. Related to Figure 6. (A) TAG and (B) DAG hydrolase activities in microsomes isolated from insect cells transduced with human CES2 (n=6-8). $^{*}p<0.05$. (C) Equal amounts of protein from membrane and cytosolic fractions were subjected to Western Blot analysis for total PKCε. Membrane to cytosol ratio is presented as arbitrary units as different exposures were used to quantify each fraction to ensure the bands remained in the linear range (n=9-10). $^{\#}$diet effect. Linear regression analysis with 95% confidence interval between (D) CES2 and (E) AADAC activities and HOMA-IR and DAG concentrations (n=17).

Supplemental Table 1: Subject Characteristics, Related to Figure 1

	**Lean**		**Obese**	
	Lipidome	ABPP	Lipidome	ABPP
M/F	4/4	4/3	5/10	5/4
Age (years)	43.4 ± 4.9	43.8 ± 5.6	36.0 ± 2.1	33.2 ± 2.5
Height (cm)	175.6 ± 4.7	174.6 ± 5.3	170.8 ± 2.1	174.1 ± 2.7
Weight (kg)	72.5 ± 6.1	71.0 ± 6.8	118.0 ± 3.2*	120.6 ± 4.2*
BMI (kg/m^2)	22.9 ± 0.8	22.7 ± 0.9	40.6 ± 1.2*	39.8 ± 1.6*
Waist (cm)	86.6 ± 4.0	87.7 ± 4.4	123.2 ± 2.6*	126.2 ± 3.2*
TG (mmol/l)	1.1 ± 0.2	1.2 ± 0.2	1.7 ± 0.3	1.8 ± 0.5
HDL (mmol/l)	1.6 ± 0.2	1.5 ± 0.1	1.2 ± 0.1*	1.2 ± 0.1
LDL (mmol/l)	2.7 ± 0.5	2.8 ± 0.5	3.3 ± 0.2	3.1 ± 0.2
Cholesterol (mmol/l)	4.8 ± 0.5	4.9 ± 0.6	5.2 ± 0.2	5.0 ± 0.2
Glucose (mmol/l)	5.9 ± 0.4	6.1 ± 0.4	5.7 ± 0.2	5.7 ± 0.1
HbA1c (mmol/mol)	31.9 ± 1.6	32.3 ± 1.8	34.2 ± 2.4	31.8 ± 1.5
Insulin (µU/mL)	7.8 ± 1.0	8.2 ± 1.1	n/a	22.3 ± 1.5*
HOMA – IR	2.0 ± 0.3	2.2 ± 0.3	n/a	5.9 ± 1.2*
ASAT (units/l)	40.6 ± 6.1	42.0 ± 6.8	n/a	44.7 ± 8.8
ALAT (units/l)	49.3 ± 9.7	52.5 ± 10.5	n/a	70.1 ± 21.5
Medication Use (n)	0	0	1[¤],1[^],1[$]	1[¤],1[^]

Data is presented as mean ± S.E.M. n/a: data not available. * p<0.05 vs. lean controls. [¤]Citalopram. [^]Bisoprolol. [$]Enalapril.

Supplemental Table 2: Western Blot Quantification, Related to Figures 3, 4, and 5.

Target	chow - GFP (n=11-13)	chow - CES2 (n=12-14)	HF - GFP (n=9-12)	HF - CES2 (n=14-17)
Figure 3B				
mouse CES2[#]	8.7 ± 0.7	7.6 ± 0.6[a]	6.3 ± 0.3	6.8 ± 0.5[a]
human CES2[#,§,X]	0.3 ± 0.0	8.4 ± 0.8*	0.4 ± 0.0	16.2 ± 1.4*
Figure 4C				
Akt[§]	7.7 ± 0.6	9.5 ± 0.5	9.0 ± 0.7	10.8 ± 0.6
P-Akt (Ser473) [#,§]	14.4 ± 2.8	23.6 ± 3.3	22.4 ± 2.2	34.4 ± 4.4*
FoxO1 (C29H4)	5.8 ± 0.5	7.5 ± 1.2	5.0 ± 0.7	7.2 ± 0.5
P-FoxO1 (Ser256) [§]	5.5 ± 0.4	6.7 ± 0.4	5.4 ± 0.4	6.7 ± 0.3*
GSK-3α/β [§]	8.4 ± 0.3	10.8 ± 0.3*	9.1 ± 0.5	11.6 ± 0.4*
P-GSK-3α/β (Ser21/9) [#,§]	1.3 ± 0.2	2.4 ± 0.3*	2.4 ± 0.3	3.3 ± 0.3*
IRS1[#]	12.3 ± 0.9	11. 1± 0.8	7.4 ± 1.0	6.5 ± 0.8
P-IRS1 (Ser307)[#,§,X]	6.7 ± 1.3	6.2 ± 1.0	6.7 ± 0.9	16.2 ± 1.8*
P-IRS1 (Tyr612)[§]	12.3 ± 1.2	10.4 ± 0.9	15.6 ± 2.5	9.4 ± 2.2
mTOR (7C10)	7.1 ± 0.2	7.4 ± 0.1	6.9 ± 0.2	6.9 ± 0.2
P-mTOR (Ser2448)[§]	8.7 ± 0.8	12.0 ± 0.9	8.6 ± 1.3	11.7 ± 1.2
Figure 5A				
CHOP[#,§]	7.0 ± 0.8	7.8 ± 0.3	10.7 ± 1.2	14.7 ± 1.0*
P-eIF2α (Ser51) [#,§]	8.7 ± 0.7	9.6 ± 0.5	6.1 ± 0.8	8.9 ± 0.5*
IκBα	11.2 ± 1.2	13. 0± 1.4	11.4 ± 1.3	12.7 ± 1.1
P-IκBα (Ser32/36)[#,§]	5.6 ± 0.3	6.8 ± 0.6	8.9 ± 1.0	12.4 ± 0.9*
IKKα/β	6.8 ± 0.2	6.9 ± 0.2	6.9 ± 0.3	7.7 ± 0.3
P-IKKα/β (Ser176/180)[§]	7.9 ± 1.1	11.7 ± 1.1	9.1 ± 1.5	14.4 ± 1.3*
JNK[#]	5.3 ± 0.3	5.7 ± 0.4	4.6 ± 0.3	4.7 ± 0.4
P-JNK (Thr183/Tyr185)[§]	5.1 ± 0.6	8.4 ± 0.7*	6.5 ± 0.7	7.6 ± 0.4
acetyl-NF-κB p65 (Lys310)[#,§]	8.0 ± 0.5	4.6 ± 0.4*	5.3 ± 0.4	3.9 ± 0.5
NF-κB p65[§]	9.9 ± 0.2	12.4 ± 0.8	9.7 ± 0.8	11.7 ± 0.9
P-NF-κB p65 (Ser536)	14.2 ± 1.4	12.0 ± 0.8	14.9 ± 1.2	12.8 ± 0.6

Data is presented as mean ± S.E.M. [a]Due to ~30% cross reactivity with human CES2 this data must be interpreted with caution. [#]diet effect,[§] virus effect, [x] interaction, *p<0.05 Bonferroni *post hoc* test.

Supplemental Methods

Primers Used

**Human**	**Forward**	**Reverse**
36b4	CCCTGAAGTGCTCGACATCA	TGCGGACACCCTCCAGAA
aadac	TGCAGGAGGGAATTTAGCTG	TGACATCTGGGTCATCAAGG
atf3	CGCTGGAATCAGTCACTGTC	TTTCTCGTCGCCTCTTTTTC
ces2	GTCTTCGCTTGTTGTGTCC	AACTTGGTCACAGGCAGACA
chop	AGCCAAAATCAGAGCTGGAA	CGAAGGAGAAAGGCAATGAC
g6pase	TGCCCCTGATAAAGCAGTTC	TCGGCTTTATCTTTCCCTGA
pepck	GTGCTTTGCTCTCAGGATGG	CCGCCAGGTACTTCTTCTCA
pgc1α	GCACCGAAATTCTCCCTTGT	GCCTCTCGTGCTGATATTCC
trib3	TGCCCTACAGGCACTGAGTA	GGCGTAGAGGAGCTGGGTA
**Mouse**	**Forward**	**Reverse**
acc1	GATGAACCATCTCCGTTGGC	CCCAATTATGAATCGGGAGTGC
acox1	TTGGAAACCACTGCCACATA	GCCAGGACTATCGCATGATT
ccl2	CCCAATGAGTAGGCTGGAGA	TCTGGACCCATTCCTTCTTG
ccl5	GCTGCTTTGCCTACCTCTCC	TCGAGTGACAAACACGACTGC
ces2a	CTCACAGCCGGCCATGT	AGATTCATTTCCTTCGCATCCT
ces2b	TCTGAGATGGTCTCCACTACG	GCAGGGATCATCTGGACAAGC
ces2c	GCTGAATGCTGGGTTCTTCG	GCTGCCTTGGATCTGTCCTGT
ces2e	CTTGTCTTTGGCTACCAGTTCG	TTGCTCCTCTTCCTCAGTGTAAGG
ces2f	TTCAGCGTTCCCATGCTCCT	CTTGAGTAAACTGGACCTATGCTG
ces2g	TCTCTGAGGTGGTTTACCAAACG	CCTCTCAGACAGCGCACCAG
ces2h	AACTGTCTACGAGGCAAAAGCG	GAGGATGTCTGGGCAGGAAGAT
cpt1a	ACGGAGTCCTGCAACTTTGT	GTACAGGTGCTGGTGCTTTTC
cxcl10	ATGACGGGCCAGTGAGAATG	ATGATCTCAACACGTGGGCA
cxcr3	CACAAGTGCCAAAGGCAGAG	AAGTCCGAGGCATCTAGCAC
emr1	TCACTGTCTGCTCAACCGTC	AGAAGTCTGGGAATGGGAGC
fasn	CCCGGAGTCGCTTGAGTAT	GGATTTGGTGGAGCCAATTA
g6pase	CGACTCGCTATCTCCAAGTGA	GTTGAACCAGTCTCCGACCA
hprt	AGCAGTACAGCCCCAAAATG	AGAGGTCCTTTTCACCAGCA
il1β	CTCATCTGGGATCCTCTCCA	GGGTCCGTCAACTTCAAAGA
pepck	CTCCTTTGGAAGCGGATATG	TGCCTTCGGGGTTAGTTATG
pgc1α	TATGGAGTGACATAGAGTGTGCT	CCACTTCAATCCACCCAGAAAG
pmp70	TGTTCAGGACTGGATGGATG	TGGCAAACTGGGGTTTATG
pparα	CCTGAACATCGAGTGTCGAA	CAGCTCCGATCACACTTGTC
scd1	CCTGCGGATCTTCCTTATCA	GCCCATTCGTACACGTCATT
srebp1c	GATGTGCGAACTGGACACAG	GCATGTCTTCGATGTCGTTCAAA
tgf1β	GGAGAGCCCTGGATACCAAC	CGCACACAGCAGTTCTTCTC
tnfα	CCACCACGCTCTTCTGTCTA	AGGGTCTGGGCCATAGAACT

Antibodies Used

Target	Catalogue #	Company
Acetyl-NF-κB p65 (Lys310) (D2S3J)	12629	Cell Signaling
Phospho-IKKα/β (Ser176/180) (16A6)	2697	Cell Signaling
Phospho-IκBα (Ser32/36)	9246	Cell Signaling
Phospho-NF-κB p65 (Ser536)	3033	Cell Signaling
Phospho-SAPK/JNK (Thr183/Tyr185) (81E11)	4668	Cell Signaling
Phospho-eIF2α (Ser51)	9721	Cell Signaling
IκBα	9242	Cell Signaling
NF-κB p65	3034	Cell Signaling
Phospho-FoxO1 (Ser256)	9461	Cell Signaling
Akt	9272	Cell Signaling
mTOR (7C10)	2983	Cell Signaling
FoxO1 (C29H4)	2880	Cell Signaling
Phospho-Akt (Ser473)	9271	Cell Signaling
Phospho-mTOR (Ser2448) (D9C2)	5536	Cell Signaling
GSK-3α/β (D75D3)	5676	Cell Signaling
Phospho-GSK-3α/β (Ser21/9)	9331	Cell Signaling
JNK	sc-571	Santa Cruz Biotechnology
IKKα/β (H-470)	sc-7607	Santa Cruz Biotechnology
GADD 153 (B-3)	sc-7351	Santa Cruz Biotechnology
Anti-phospho-IRS1 (Ser307 mouse)	05-1087	Merck Millipore
Anti-IRS1	06-248	Merck Millipore
Phospho-IRS1 pTyr612	44-816G	Thermo Fisher Scientific
Human CES2	PA5-34842	Thermo Fisher Scientific
Mouse Carboxylesterase 2/CES2	AF5280	R&D Systems
PKCε	610086	BD Biosciences
